# Impact of noradrenaline, adrenergic receptors, and radical formation on cardiac mitochondria in resuscitated swine

**DOI:** 10.1186/s40635-026-00925-1

**Published:** 2026-06-09

**Authors:** Nadja Abele, Tamara Merz, Fabian Zink, Andrea Hoffmann, Michael Gröger, Sandra Kress, Stefanie Kranz, Andrea Seifritz, Vittoria Passarelli, Franziska Münz, Maximilian Feth, Magnus Scheer, Ohad Sharon, Melanie Hogg, Eva-Maria Wolfschmitt, Ulrich Wachter, Tanja Schulz, Enrico Calzia, Nicolas Fage, Christiane Waller, Pierre Asfar, Peter Radermacher, Thomas Kapapa

**Affiliations:** 1 Institut Für Anästhesiologische Pathophysiologie Und Verfahrensentwicklung, Universitätsklinikum, Ulm, Germany; 2 Klinik Für Anästhesiologie Und Intensivmedizin, Universitätsklinikum, Ulm, Germany; 3 Klinik Für Anästhesiologie, Intensivmedizin, Schmerztherapie Und Notfallmedizin, Bundeswehrkrankenhaus, Ulm, Germany; 4 Klinik Für Neurochirurgie, Bundeswehrkrankenhaus, Ulm, Germany; 5 Klinik Für Neurochirurgie, Universitätsklinikum, Ulm, Germany; 6 Département de médecine intensive—réanimation et médecine hyperbare, Centre Hospitalier Universitaire, Angers, France; 7 Klinik Für Psychosomatische Medizin Und Psychotherapie, Klinikum Nürnberg, Campus Nord, and Paracelsus Medizinische Privatuniversität, Nuremberg, Germany

**Keywords:** High resolution respirometry, Immunohistochemistry, Mitochondrial respiratory complexes, Electron spin resonance, Superoxide anion, Alkaline comet assay, Isoprostanes, Nitrate/nitrite, Nitrotyrosine, Troponin

## Abstract

**Background:**

Equivocal data exist on whether noradrenaline during resuscitation from circulatory shock affects tissue mitochondrial respiration. Therefore, we examined the relation between myocardial mitochondrial respiration and plasma noradrenaline concentrations during continuous i.v. noradrenaline.

**Methods:**

After anesthesia and (neuro)surgical instrumentation, pigs underwent two hours of subdural blood injection and subsequent systemic blood removal, followed by fluid resuscitation, re-transfusion of shed blood, and noradrenaline to maintain cerebral perfusion pressure at pre-shock levels. Before, immediately after the challenge, and after 48 h of resuscitation, we measured whole blood superoxide anion levels (electron spin resonance) and DNA strand breaks ("tail moment" in the comet assay), and plasma noradrenaline (HPLC/tandem mass-spectrometry), NOx (chemoluminescence), isoprostane and troponin (ELISA) concentrations. Immediate *post mortem* myocardial samples were analyzed for mitochondrial respiration (high-resolution respirometry for Complex I/II activity, oxidative phosphorylation, and maximal uncoupled electron transfer capacity) as well as β_1_-/β_2_-adrenergic receptor and mitochondrial complex I/II expression, and nitrotyrosine formation (immunohistochemistry).

**Results:**

At 48 h of resuscitation, plasma noradrenaline concentrations were 200-fold those at baseline, while NOx levels had doubled. Myocardial mitochondrial respiration was unrelated to noradrenaline, NOx, or nitrotyrosine, but directly related to both the β_1_- and β_2_-adrenoceptor expression (oxidative phosphorylation: r = 0.466, p = 0.059 and r = 0.750, p = 0.0005, respectively; maximal uncoupled electron transfer capacity: r = 0.514, p = 0.035 and r = 0.772, p = 0.0003, respectively). Oxidative phosphorylation was inversely related to the plasma troponin-I levels (r = -0.479, p = 0.060). While plasma NOx levels correlated with plasma noradrenaline levels (r = 0.519, p = 0.033), whole blood superoxide anion levels and cardiac nitrotyrosine formation did not.

**Conclusions:**

In a long-term, resuscitated porcine model of acute subdural hematoma and hemorrhage, plasma NOx levels correlated with noradrenaline plasma levels, whereas mitochondrial respiration and other markers of radical formation or oxidative/nitrosative stress were unrelated to noradrenaline concentrations. β-adrenergic receptor expression was also unrelated to noradrenaline, while mitochondrial respiration was directly related to both the β_1_- and β_2_-adrenoceptor expression. Our results suggest that the impaired mitochondrial respiration reported during septic shock for skeletal muscle may be absent in the heart during continuous i.v. noradrenaline for resuscitation from trauma-and-hemorrhage. Nevertheless, cardiac tissue injury is associated with reduced mitochondrial respiration.

**Supplementary Information:**

The online version contains supplementary material available at 10.1186/s40635-026-00925-1.

## Background

Noradrenaline (NoA) is the first line drug choice for the management of circulatory shock-related hypotension, because due to its combined α- and β-adrenergic activity, it exerts both vasopressor as well as positive ino- and chronotropic effects. However, in animal experiments high-dose NoA was associated with mitochondrial dysfunction and excess formation of reactive oxygen species (ROS): in healthy dogs, continuous i.v. infusion (2 and 5 μg/kg/min over 5 h) resulted in depression of cardiomyocyte mitochondrial respiration [1], while in rats, a 3 mg/kg i.p. bolus of NoA was associated with myocardial tissue ascorbic acid depletion, increased malondialdehyde levels and a several-fold fall in the ratio of reduced to oxidized glutathione, an indicator of cellular redox status and oxidative stress [2]. In patients with septic shock, skeletal muscle NOx content, an indicator of increased nitric oxide (NO) formation, and mitochondrial complex I activity were directly and inversely related, respectively, to the NoA infusion rates needed to achieve target hemodynamics [3].

Long-term, i.e., over one to eight weeks [4–7], high-dose (200–300 μg/kg/h in rats [4, 6, 7], 30 μg/kg/h in dogs [5]) NoA administration also reduced both myocardial β_1_-adrenocepor receptor expression and receptor sensitivity, resulting in decreased responsiveness to β-agonists; in contrast, short-term infusion, i.e., for two hours only, was also associated with receptor desensitization, whereas receptor density was not yet affected. Interestingly, any effect of NoA infusion may be dose-dependent in this context: in dogs, even after three months, a continuous i.v. NoA infusion, which had not produced arterial hypertension (≈ 3 μg/kg/h), both β_1_-adrenocepor receptor expression and responsiveness were even increased [8].

So far, most experimental data on a possible impact of supra-normal plasma NoA concentrations resulting from exogenous administration on myocardial β-receptor density and/or mitochondrial respiration originate from studies lacking intensive care. Studies integrating standard intensive care measures produced equivocal findings: During porcine endotoxemia, continuous i.v. NoA to achieve a mean arterial pressure ≈ 95 mmHg increased liver mitochondrial respiratory activity; however, heart tissue was not studied [9]. Ex vivo NoA stimulation of liver and skeletal muscle specimens from pigs with fecal peritonitis did not reveal any changes in mitochondrial respiration activity; again, no data on heart tissue measurements was reported [10]. In another study of this group, allocating swine with fecal peritonitis-induced septic shock to either "no resuscitation", "low", or "high" mean arterial pressure targets resulted in significantly higher NoA requirements in the "high" target group. Liver and skeletal muscle mitochondrial respiratory activity did not differ between the two NoA treatment regimens; again, no data on heart mitochondria was presented [11]. When comparing NoA with angiotensin to achieve comparable systemic hemodynamics in this model, kidney mitochondrial activity was slightly lower in the angiotensin-treated animals, whereas liver and, in particular, heart mitochondrial respiratory activity did not differ [12].

Finally, immediately prior to the present experimental series, we showed in naïve swine from the same strain and comparable with respect to age, sex and body weight, but which only underwent induction of anesthesia and light, short-term surgical instrumentation that variations of the plasma noradrenaline concentrations without exogenous NoA administration, i.e., within the physiological range, were inversely related to myocardial mitochondrial respiratory activity and β_2_-adrenoceptor expression, whereas there was no significant relation to mitochondrial respiratory complex expression, oxygen radical production or to markers of oxidative stress [13]. Hence, given the equivocal data on NoA effects on myocardial mitochondrial respiratory activity, the present study tested the hypothesis whether the above-mentioned inverse relationship between myocardial mitochondrial respiratory activity and plasma noradrenaline concentrations within the *physiological* range could be confirmed for *pharmacological* NoA plasma levels, i.e., during continuous i.v. NoA to achieve systemic hemodynamic targets. For this purpose, we used our clinically relevant, long-term porcine model of combined acute subdural hematoma and hemorrhage with subsequent resuscitation [14, 15]. We investigated putative relations between immediate *post mortem* cardiac tissue mitochondrial respiratory activity ("high resolution respirometry") and NoA plasma concentrations (HPLC/tandem mass-spectrometry). In addition, we evaluated the possible role of adrenergic receptor and mitochondrial respiratory complex expression (immunohistochemistry) and oxygen (electron spin resonance) and nitrogen (chemoluminescence) radical formation as well as markers of oxidative (comet assay for DNA strand breaks; isoprostanes for lipid peroxidation) and nitrosative stress (immunohistochemistry for tissue nitrotyrosine formation).

## Methods

In order to comply with the "3R principle" for animal experimentation, the present study made use of our well-established, long-term, porcine model of combined acute subdural hematoma plus hemorrhagic shock with subsequent resuscitation [14, 15] rather than investigating, e.g., cardiogenic or pure hemorrhagic shock. In this model high-dose NoA infusion rates are required to maintain hemodynamic targets so that supra-physiological NoA concentrations are always present during the resuscitation period. A total of 39 animals were used, 2 of which had to be excluded due to complications during the instrumentation period: one animal died from an uncontrollable hemorrhagic complication during the surgical instrumentation, a second one presented with acute respiratory failure immediately after endotracheal intubation that had been caused by pneumonia as diagnosed upon necropsy. Another 16 animals were excluded from the analysis, because they had to be euthanized prior to the pre-determined end of the monitoring and treatment period, i.e., at 48 h of ICU care, because cerebral perfusion pressure (CPP, i.e., the difference between mean arterial (MAP) and intracranial (ICP) pressure) could not be maintained ≥ 50 mmHg despite the maximum dose of vasopressors (limited to a heart rate of 160 per minute to prevent myocardial injury induced by tachycardia), or acute anuric kidney failure with consecutive hyperkalemia (blood potassium > 6 mmol/L) and subsequent cardiac arrhythmia. Therefore, the data presented are from 21 young, sexually mature human-sized (median (interquartile range): age 150 (146; 164) days); body weight 79 (75; 86) kg) German Large White pigs of either sex (10 females, 11 males). The individual animals studied were part of a larger experimental series comprising a "control" group (n = 21; 11 females, 10 males) and a group exposed to "early life stress (ELS)" (n = 16, females and males each n = 8). Animals of the control group had been weaned on day 28–35 after birth, which corresponds to the weaning period regularly used for swine husbandry. In contrast, ELS swine had already been weaned on day 21 after birth. This weaning time point approach has already been used previously [13, 16], because (1) it represents the earliest time point for swine weaning described in the Federal German regulations on farm animal husbandry and (2) since we aimed to avoid any pathological clinical symptoms associated with earlier (at day 15–19) weaning of piglets in other ELS models, e.g., diarrhea, weight loss, and/or intestinal mucosal barrier dysfunction [17–20]. In order to minimize inter-individual differences with respect to age and development as far as possible, pairs of control and ELS animals had been taken from the same litter; in fact, ELS and control swine did not show any difference with respect to growth, body weight, and behavior. The animals had been obtained from an agricultural production specified in breeding and rearing of piglets (Schweinezucht Kugler Gmbh & Co. Kg, Ostrach, Germany). This is also where the weaning was performed. After transfer of the 5–6 months old pigs to our facility, they were kept separated by sex in separate boxes, but in contact to the neighboring animals. They had regular run and were provided activity by the animal caretaker. They received the same fodder as before, provided by the breeder, additionally they had ad libitum access to hay and straw. Male and female animals were kept under the same conditions. Male animals had not been castrated to allow for higher translational relevance and more distinct evaluation of possible sex-dependent effects. At the breeding facility, regular health monitoring by the "Schweinegesundheitsdienst" (pig health service) and the "Tierseuchenkasse" (animal plague office) was performed.

### Anesthesia and surgery

The procedures for anesthesia and surgical instrumentation have been described in detail previously [14, 15]. The animals had free access to water until immediately before the pre-medication. Prior to instrumentation, the animals were sedated by intramuscular injection of azaperone (200 mg) and midazolam (45–60 mg). An intravenous catheter was established in an auricular vein. Anesthesia was induced by intravenous injection of propofol (1.5–2 mg/kg) and ketamine (100 mg). Subsequently, the pigs were endotracheally intubated and mechanically ventilated (ventilator settings: tidal volume 8 mL/kg, respiratory rate 10–16 breaths per min adapted to achieve an arterial PCO_2_ (PaCO_2_) of 35–40 mmHg, inspiratory/expiratory (I/E) ratio of 1:1.5, fraction of inspiratory oxygen (F_I_O_2_) of 0.3, positive end-expiratory pressure (PEEP) 10 cmH_2_O to minimize general anesthesia-induced atelectasis formation). Anesthesia was maintained by continuous infusion of propofol (10 mg/kg/h), midazolam (1 mg/kg/h), and remifentanil (initial bolus: 5 mg/kg, followed by 15–20 μg/kg/h). During laryngoscopy, a tube was advanced into the stomach for decompression and drainage of secretion. To maintain fluid balance, a balanced electrolyte solution (10 mL/kg/h, Jonosteril 1/1®, Fresenius Kabi) was infused. After surgical exposure, a 4-lm venous catheter (7 Fr, Teleflex, Reading, USA) was placed in the right iliac vein via a 10-F sheath (Super Arrow-Flex Percutaneous Sheath Introducer Set, Teleflex) to measure the central venous pressure, to return the shed blood, and to administer the required medication. The resulting central venous pressure should be interpreted with caution, because the tip of the catheter was located in the inferior vena cava, and due to the influence of the applied PEEP. Moreover, another 10-F sheath was placed in the left femoral artery for blood sampling and induction hemorrhage shock by passive removal of blood as described below. In addition, for continuous measurement of cardiac output, a 5-F PiCCO catheter (PULSION Medical Systems, Munich, Germany) was inserted into the right femoral artery. A midline mini-laparotomy was performed to insert a catheter into the urinary bladder. Subsequently, the pigs were put in a prone position for the neurosurgical instrumentation consisting of a craniotomy over both parietal cortices. For the induction of acute subdural hematoma (ASDH) later on, the dura was opened and a ventricular catheter (9F, Neuromedex, Hamburg, Germany) was inserted approximately 5 mm into the subdural space. The catheter was placed into the left hemisphere. Thereafter, multimodal brain monitoring probes (Neurovent-PTO, Raumedic AG, Helmbrechts, Germany) were inserted approximately 10–15 mm into the parenchyma of both hemispheres. These multimodal brain monitoring probes were used for the measurement of intracranial pressure (ICP) and both brain tissue temperature and O_2_ partial pressure (P_bt_O_2_). After equilibration of both catheters and stabilization of P_bt_O_2_, recording was started according to the manufacturer’s instructions. Bone wax was used to close the burr holes as well as to fix the multimodal brain monitoring probes. Body temperature was assessed by a rectal probe, and during the resuscitation period animals were kept at a temperature of 38–39 °C. After the initiation of resuscitation, the temperature was controlled to maintain brain normothermia. If the brain temperature was ≥ 39 °C, the pigs received external cooling by ice-cold water-filled bags.

### Experimental protocol

After the instrumentation and a subsequent resting period of approximately 90 min, the experimental protocol was similar to our above-mentioned previous studies [14, 15]. The protocol comprised a period of combined ASDH and hemorrhage (2 h) and a subsequent resuscitation period of 48 h. To mimic the typical clinical situation where resuscitation procedures are initiated with a certain delay after trauma-and-hemorrhage, the fluid infusion rate was reduced to 20 mL/h, the ventilator settings were changed to a tidal volume of 8 mL/kg, PEEP 0 cm H_2_O, I/E ratio 1:2, F_I_O_2_ 0.21. To initiate ASDH, 0.1 mL/kg of autologous blood was injected over 15 min through the subdural catheter using an automated syringe pump. This approach was chosen (1) based on the rationale that in previous studies on porcine ASDH, the injection of a blood volume approximating 10% of the intracranial volume represents the threshold for supra-tentorial volume tolerance [21, 22] and (2) because it had been successfully used in our previous studies using this model [14, 15]. Following the induction of ASDH, hemorrhage was initiated by passive removal of blood for 30 min using the large bore arterial catheter targeting 30% of the calculated blood volume. For that purpose, the target blood volume to be withdrawn (in [g]) was calculated according to following equation:

$$0.{3}\cdot{ 8}0 \, \left[ {{\mathrm{mL}}/{\mathrm{kg}}} \right] \, \cdot{\text{ body weight }}\left[ {{\mathrm{kg}}} \right] \, \cdot{ 1}.{1 }\left[ {{\mathrm{g}}/{\mathrm{mL}}} \right],$$

assuming that total blood volume and blood density were 8% of body weight and 1.1 g/mL, respectively [23, 24].

The removed blood was stored at room temperature in acid-citrate-dextrose solution until re-transfusion. Blood removal was decelerated or interrupted, or small volumes of blood were re-transfused as necessary to maintain cerebral perfusion pressure (CPP), i.e., the difference between mean arterial pressure (MAP) and ICP, ≥ 50 mmHg. This regimen was chosen to prevent irreversible brain damage based on our previous experiences [14, 15]. After two hours of combined ASDH (15 min) and hemorrhage (105 min), resuscitation was initiated. Resuscitation included re-transfusion of the shed blood within 30 min, fluid resuscitation (10 mL/kg/h Jonosteril, reduced to 5 mL/kg/h if central venous pressure > 16 mmHg and/or occurrence of oligo-/anuria) as well as by continuous i.v. NoA titrated to maintain CPP at pre-shock levels and ≥ 60 mmHg (the latter as referred to in current guidelines [25]). Upon initiation of resuscitation, the baseline ventilator settings were set to tidal volume 8 mL/kg, respiratory rate 12–20 breaths per min to maintain PaCO_2_ of 35–40 mmHg, I/E ratio of 1:1.5, and PEEP 10 cm H_2_O. The F_I_O_2_ was titrated to maintain normoxemia (PaO_2_ = 80–120 mmHg). After 48 h of intensive care, following further deepening of the anesthesia, the pigs were sacrificed by injection of potassium chloride. Immediately *post mortem*, left ventricular heart apex specimens were harvested for further tissue analyzes.

### Measurements and calculations

Sampling of the presented parameters was carried out 30 min before the start of ASDH and hemorrhage (referred to as "Baseline" in tables), at the end of the 2 h of ASHD and hemorrhage period ("2 h ASDH + hemorrhage"), and after 48 h ("48 h ICU"). Arterial sampling was chosen to prevent potential interferences with the administration of drugs and infusions, to reduce the need for additional venous instrumentation, and to ensure rapid sampling. In addition, this sampling method can be considered to analyze the systemic levels of the analytes, which are distributed to all organs (and thus potentially affecting them) and to result in well-mixed samples affected by all organs and tissues. Hemodynamics, arterial blood gas tensions, acid–base status, glucose, and lactate levels were determined as previously described [14–16]. In brief, blood gas analysis, total hemoglobin, electrolyte, glucose, and lactate levels were measured using a standard blood gas analyzer (ABL 800 Flex, Radiometer GmbH, Krefeld, Germany).

### Catecholamines, parameters of oxygen radical and nitric oxide formation, oxidative stress and cardiac injury

Plasma catecholamine (adrenaline, noradrenaline) levels were determined after centrifugation of whole blood samples in Li^+^-heparin coated, stabilizer-primed (20 μL/mL blood containing 0.2 M reduced glutathione and 0.2 M ethylenglycol-bis(aminoethylether)-N,N,N0,N0-tetra-acetic acid (EGTA), both Carl-Roth, Karlsruhe, Germany) tubes using liquid-chromatography/tandem-mass-spectrometry (LCMS/MS) (external analysis by Dr. Eberhard & Partner, Dortmund, Germany). Sample values below the detection limit were assigned the value of the detection limit of 15 pg/mL [13].

Superoxide anion radical (O_2_^⋅^¯) concentrations in whole blood samples were determined by electron spin resonance as described previously [13, 16]. Immediately after sampling arterial blood to a LiHep-monovette (Sarstedt), 25 µL blood were mixed with 25 µL spin probe solution (400 µM CMH (1-Hydroxy-3-methoxycarbonyl-2,2,5,5-tetramethylpyrrolidine, all ESR reagents from Noxygen, Elzach, DE) in Krebs-HEPES-Buffer containing 25 µM deferoxamine and 5 µM diethyldithiocarbamate). The mixture was transferred to a 50 µL glass capillary and incubated for 5 min at 37 °C (Bio-III, Noxygen) before measurement at 37 °C as described previously [13] with an EMXnano ESR spectrometer (Bruker BioSpin, Billerica, MA, USA). Blank values were obtained by measuring the spin probe solution with 25 µL of the Krebs-HEPES-Buffer and subtracted from the blood sample values. Quantification was possible by comparison of the sample amplitudes with those of a carboxy-proxyl standard dilution series.

As marker of O_2_^⋅^¯-induced oxidative stress, whole blood DNA single strand-breaks were quantified as "tail moment" using single cell gel electrophoresis (alkaline version of the "comet assay") [26] adapted for swine blood as described previously [13]. For this purpose, 5 μL whole blood was mixed with 120 mL LMP-Agarose (37 °C) and applied on a slide. Slides were stored in lysis-buffer for 2 days at 4 °C. Briefly after lysis, the cells were de-naturated with alkali (electrophoresis buffer pH 13) for 40 min, followed by electrophoresis for 40 min at 25 V and 300 mA. Slides were stained with 50 μL ethidium bromide and evaluated by image analysis (Comet Assay II, Perceptive Instruments, Haverhill, UK). Results are shown as mean tail moment (percentage of DNA in the tail × tail length) of 50 randomly selected nuclei per slide (two slides each per measurement) according to the image analysis software (Comet Assay II V1.02).

Plasma 8-isoprostane levels were measured as a marker of lipid peroxidation as described previously [13]. For this purpose, LiHeparin blood samples were centrifuged at 4 °C, and the obtained plasma was stored at − 80 °C until analysis. After purification with C-18 solid-phase extraction (Supelclean LC-18, Sigma-Aldrich), plasma samples were analyzed with the Cayman Chemical assay kit (8-isoprostane ELISA kit 516,351, Cayman Chemicalm Ann Arbor, MI, USA).

As a surrogate for nitric oxide (NO) formation, plasma concentrations of the stable NO metabolites nitrite and nitrate (NO_2_¯ + NO_3_¯; "NOx") were measured as described previously using a chemoluminescence analyzer for gaseous and liquid samples after reduction of NOx to NO with vanadiumchloride (NOA 280 NO Analyzer, Sievers Medical Instruments [27]).

As a marker of cardiac injury, plasma troponin-I levels were measured using a commercially available species-specific ELISA kit (High Sensitivity Pig Cardiac Tropopnin-I ELISA, Cat. No. CTNI-9-HSP, Life Diagnostics, West Chester, PA) according to the manufacturer’s manual.

For technical reasons whole blood O_2_^⋅^¯ and comet assay analyzes as well as plasma catecholamine, isoprostane, NOx and troponin-I concentrations were available in only 17 of the 21 swine studied.

### Tissue mitochondrial respiration

Heart tissue mitochondrial respiration was measured by high-resolution respirometry using the Oxygraph-2 K® (Oroboros Instruments, Innsbruck, Austria) as described previously [13]. This device allows for simultaneous recording of the O_2_ concentration in two parallel chambers calibrated for 2 mL of respiration medium MiR05. This medium is composed of 110 mMD-Sucrose (Sigma Aldrich, St. Louis, MO, USA), 60 mM K-Lactobionate (Sigma Aldrich, St. Louis, MO, USA), 0.5 mM ethylene glycol tetra acetic acid (Sigma Aldrich, St. Louis, MO, USA), 1 g/L bovine serum albumin free from essentially fatty acids (Sigma Aldrich, St. Louis, MO, USA), 3 mM MgCl_2_ (Scharlau, Hamburg, Germany), 20 mM taurine (Sigma Aldrich, St. Louis, MO, USA), 10 mM KH_2_PO_4_ (Merck, Darmstadt, Germany), and 20 mM HEPES (Sigma Aldrich, St. Louis, MO, USA), adjusted to pH = 7.1 with KOH and equilibrated with 21% O_2_ at 37 °C. Heart tissue homogenates containing 0.75 mg_tissue_/mL of respiration medium were filled into both chambers and continuously stirred at 750 rpm. Closing the chambers by gently pushing down the stoppers started the continuous recording of mitochondrial respiration, which was quantified in terms of O_2_ flux (*J*O_2_) based on the rate of change of the O_2_-concentration in the chambers normalized for tissue weight. Once the chambers were sealed, specific analysis of mitochondrial respiratory function was achieved by sequential injections of mitochondrial substrates and inhibitors into the respiration medium. Recording of measurements started after achieving a stable *J*O_2_-signal. The maximum respiratory capacity in the coupled state (OxPhos) was determined after the addition of 2 mM malate, 10 mM glutamate, 5 mM ADP, 5 μM cytochrome c, 10 mM pyruvate, 1 mM octanoyl-carnitine, and 10 mM succinate, and the maximum respiratory capacity in the uncoupled state (ETC) was measured after addition of 1 μL of 1 mM Carbonyl cyanide-*p*-trifluoromethoxyphenylhydrazone (FCCP), followed by repetitive titration of 0.5 mM FCCP. In addition, activity of the mitochondrial Complexes I and II in the coupled and uncoupled state, respectively, were recorded. The data shown is normalized for tissue wet weight.

### Immunohistochemistry for target tissue proteins

Immunohistochemistry was used to quantify myocardial expression of the β_2_- and β_1_-adrenoreceptors, subunits of the mitochondrial respiration complexes I–IV, and formation of nitrotyrosine as a marker of tissue oxidative and nitrosative stress. Immunohistochemistry was chosen, because (1) it is well established in the literature that densitometric analysis of colorimetric immunohistochemical staining is as acceptable a method as Western blotting for protein measurement [28], (2) we had previously established the immunohistochemistry protocols for porcine cardiac tissue specimens [13], (3) we had obtained highly significant correlations between the densitometric values and those obtained by Western blotting [29], and (4) in contrast to Western blotting, the immunohistochemistry evaluation of the tissue allows identification of the physical topography and protein expression in different cell types within the tissue specimen. Immunohistochemistry of heart specimens was performed as previously described [13, 30]. Immediate post mortem left-ventricular cardiac samples were fixed in 10% formalin for 6 days, dehydrated, and embedded in paraffin blocks. Paraffin Sections (3–5 μm) were cut, de-paraffinized in xylene, and rehydrated in a graded series of ethanol (100% Ethanol (1 min), 100% Ethanol (5 min), 90% Ethanol (3 min), 70% Ethanol (5 min)) and deionized water. Heat-induced antigen retrieval was performed by heating up the slides in a microwave to a rolling boil for 2 times in 10 mM citrate solution (pH 6). After cooling back to room temperature, the slides were blocked for 20 min with 10% normal goat serum (Jackson ImmunoResearch Laboratories, Inc., West Grove, PA, UK) before incubating for 1 h with the following primary antibodies: β_1_-adrenergic receptor (ADRB2 rabbit polyclonal antibody (proteintech 28,323-1AP), β_2_-adrenergic receptor (ADRB2 rabbit polyclonal antibody, proteintech 29,864-1-AP), NADH dehydrogenase (ubiquinone) 1 beta subcomplex 8 (NDUFB8 rabbit polyclonal antibody, proteintech 14,794-1-AP), succinate dehydrogenase complex subunit A (SDHA rabbit polyclonal antibody 14,865-1-AP), nitrotyrosine (Anti-Nitrotyrosine polyclonal antibody, Millipore, MA, USA, AB5411).

Primary antibody detection was performed by a Dako REAL detection system (anti-mouse, anti-rabbit; alkaline phosphatase conjugated) and visualized with red chromogen (Dako REAL; Dako, Agilent Technologies, Santa Clara, CA, USA) followed by counterstaining with hematoxylin (Sigma). Washing steps (TBS 1 min, TBS Tween 3 min, TBS 1 min) were performed after primary antibody incubation (1 h), Dako REAL, Biotinylated Secondary Antibodies incubation (30 min), Dako REAL Streptavidin Alkaline Phosphatase (AP) incubation (30 min), and Dako REAL red chromogen. A Zeiss Axio Imager A1 microscope with a 10 × objective was used for visualization of the slides. Two representative 800,000 μm^2^ sections per slide were graded for quantification of the red chromogen using the Zen Image Analysis Software 3.0 blue edition (Zeiss, Oberkochen, Germany). All primary antibodies were titrated to their optimal dilutions within the range recommended by the manufacturer. Antibody specificity had been confirmed in NCBI BLAST searches (courtesy of the U.S. National Library of Medicine, https://blast.ncbi.nlm.nih.gov/Blast.cgi, November 2023). We compared immunogen sequences or the whole-length immunogen protein (if the sequences were not available from the manufacturer) of the antibodies used to the *Sus scrofa* database. Immunohistochemistry results are presented as mean densitometric sum read, a dimensionless parameter integrating the % of positive-stained area and staining intensity [31].

### Data analysis

For the underlying animal experiment, a power analysis (power 0.8, α = 0.05) based on the primary criterion "*Time course of the Veterinary Modified Glasgow Coma Scale (VMGCS)”* [31] had yielded an n = 11 each for the 4 groups male/female and presence/absence of ELS, respectively. The data shown represents the results obtained after a pre-planned interim analysis of n = 8–9 in each group. Since there was neither any effect of the group assignment to the "control" and "early life stress (ELS)" groups nor any sex-specific effect on plasma catecholamine concentrations, blood markers of ROS production or oxidative stress, nor on tissue mitochondrial respiration or protein expression, we performed a pooled analysis of all animals. All correlation analyzes refer to the 17 swine, for which whole blood O_2_^⋅^¯ and comet assay analyzes as well as plasma catecholamine, isoprostane, NOx and troponin-I concentrations were available.

All data are presented as median (interquartile range). Time-dependent within-group differences were tested using a Kruskal–Wallis rank sum test for multiple comparisons and a subsequent Dunn’s test for comparison with the "Baseline" data (see Tables 1, 2 and supplemental Table 1). Correlation coefficients were calculated according to Pearson for linear modeling. All statistical analyses were carried out with Origin 2019b (9.6.5) (OriginLab Corporation, Northampton, MA, USA).

**Table 1:** Hemodynamic, gas exchange, acid–base status, and metabolic parameters before ("Baseline"), immediately after combined acute subdural hematoma and hemorrhage ("2 h ASDH + hemorrhage"), as well as at 48 h of intensive care ("ICU")

	Baseline	2 h ASDH + hemorrhage	48 h ICU
Blood temperature (°C)	36.9 (36.6; 37.5)	37.4 (37.1; 38.1)	38.7 (37.7; 39.0)^#^
Heart rate (1/min)	70 (60; 82)	133 (122; 146)^#^	70 (65; 83)
Mean arterial pressure (mmHg)	100 (91; 110)	60 (56; 79)^#^	108 (99; 122)
Central venous pressure (mmHg)	7 (5; 12)	5 (3; 6)^#^	9 (5; 12)
Cardiac output (L/min)	7.0 (6.0; 8.2)	5.9 (5.6; 5.9)	8.1 (7.4; 9.9)
Total hemoglobin (g/dL)	9.9 (9.2; 10.4)	8.9 (8.7; 9.2)	8.9 (8.5; 9.6)
Arterial PO_2_ (mmHg)	77 (69; 81)	67 (56; 86)	96 (91; 99)^#^
Arterial PCO_2_ (mmHg)	37 (35; 39)	37 (34; 41)	39 (35; 41)
Arterial pH	7.44 (7.42; 7.47)	7.45 (7.44; 7.49)	7.46 (7.42; 7.49)
Arterial base excess (mmol/L)	1.4 (0.5; 2.4)	2.3 (1.6; 4.0)	3.4 (1.3; 4.5)^#^
Arterial lactate (mmol/Ll)	2.3 (1.8; 2.8)	2.1 (2.0; 2.5)	0.7 (0.6; 1.0)^#^
Arterial glucose (mg/dL)	91 (83; 99)	76 (57; 95)	81 (74; 88)

Data is presented as median (interquartile range), ^#^p < 0.05 vs. "Baseline"

**Table 2:** Plasma noradrenaline, adrenaline, troponin-I, isoprostane and NOx levels as well as whole blood superoxide anion (O_2_^⋅^¯) concentrations and "tail moment" in the "comet assay" before ("Baseline"), immediately after combined acute subdural hematoma and hemorrhage ("2 h ASDH + hemorrhage"), and at 48 h of intensive care ("ICU") or at the time of experiment termination

	Baseline	2 h ASDH + hemorrhage	48 h ICU
Noradrenaline (ng/mL)	0.08 (0.04; 0.19)	0.14 (0.09; 0.26)	15.00 (12.21; 23.30)^#^
Adrenaline (pg/mL)	160 (96; 430)	32 (16; 38)^#^	15 (15; 15)^#^
Troponin-I (ng/mL)	0.05 (0.01; 0.05)	0.05 (0.04; 0.08)	0.13 (0.05; 0.31)^#^
O_2_^⋅^¯ (µM)	5.6 (5.2; 6.2)	4.4 (4.0; 4.8)^#^	4.6 (4.1; 5.1)^#^
Isoprostane (pg/mL)	75 (52; 106)	68 (46; 89)	60 (46; 129)
NOx (µM)	43 (36; 55)	41 (37; 55)	90 (72; 108)^#^
Tail moment	0.14 (0.11; 0.15)	0.15 (0.12; 0.17)	0.14 (0.14; 0.19)

Data is presented as median (interquartile range), ^#^p < 0.05 vs. "Baseline". Adrenaline concentrations below the detection limit are reported as 15 pg/mL. For technical reasons, whole blood O_2_^⋅^¯ and "tail moment" data as well as plasma catecholamine, isoprostane and NOx concentrations were available in only 17 of the 21 swine studied

## Results

### Systemic and cerebral hemodynamics, gas exchange and metabolic homeostasis

Table 1 summarizes the parameters of hemodynamics, gas exchange, acid–base status, and metabolism at the three measurement time points, i.e., before and immediately after combined acute subdural hematoma and hemorrhage (ASDH + hemorrhage) as well as at 48 h of intensive care. The corresponding data on brain tissue temperature as well as parameters of brain perfusion (ICP, CPP) and oxygenation (P_bt_O_2_ brain tissue oxygen partial pressure) are shown in the supplemental Table 1. According to the protocol, at 2 h of ASDH + hemorrhage, animals presented with significantly lowered mean arterial and central venous pressures, cardiac output, and arterial PO_2_. In order to maintain CPP ≥ 50 mmHg (see supplemental Table 1) as imposed by the experimental protocol, we could only remove a median (IQR) of 23 (17; 26) % of the calculated blood rather than the 30% targeted. Upon the start of resuscitation and during the total ICU care period, all animals needed continuous i.v. NoA to achieve the target values for MAP and CPP, respectively. At the end of the experiment all parameters recorded had returned to pre-challenge levels, except for both significantly higher arterial PO_2_ and base excess values as a result of the F_I_O_2_ of 0.25–0.35 needed to maintain the PaO_2_ target of 80–120 mmHg (see “experimental protocol”), and the use of acetated Ringer’s solution as a maintenance fluid, respectively.

### Catecholamines and systemic markers of oxidative and nitrosative stress

**Fig. 1:**
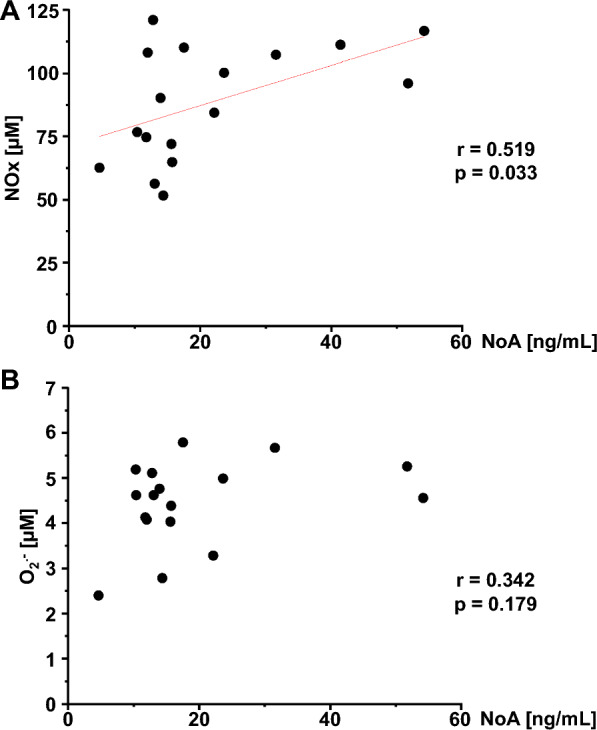
Plasma NOx levels (µmol/L [µM]) (**a**) and whole blood superoxide anion O_2_^⋅^¯ concentrations (µmol/L [µM]) (**b**) and at the end of the experiment, i.e., at 48 h of ICU care, plotted as a function of the simultaneously obtained plasma noradrenaline (NoA) levels ([ng/mL]). There was a significant linear correlation between NOx (r = 0.519, NOx = 0.8 · NoA + 71.3, p = 0.033) and NoA concentrations, while no significant relation was found between O_2_^⋅^¯ (r = 0.342, p = 0.179) and the NoA levels

Table 2 summarizes the results of the plasma catecholamine, isoprostane, NOx, and troponin-I levels as well as the whole blood superoxide anion (O_2_^⋅^¯) concentrations and "tail moment" in the "comet assay" at the three measurement time points, i.e., before and immediately after combined acute subdural hematoma and hemorrhage (ASDH + hemorrhage), as well as at 48 h of intensive care. At the end of the 2-h period of ASDH + hemorrhage, plasma NoA and troponin-I levels had increased significantly, whereas isoprostane and NOx concentrations as well as the "tail moment" in the "comet assay" remained unchanged. Plasma adrenaline and whole blood O_2_^⋅^¯ levels even significantly decreased. All animals needed continuous i.v. NoA (median (IQR): 0.78 (0.59; 1.05) µg/kg/min) to maintain target hemodynamics throughout the whole period of "ICU"-care, and, consequently, NoA plasma levels showed a progressive increase by two orders of magnitude at the end of the experiment. Supplemental Fig. 1 shows that while the individual, *terminal* NoA concentrations were directly related to the *mean* individual NoA infusion rate needed to maintain target hemodynamics (r = 0.638, p = 0.006), this mean NoA infusion rate did not allow to predict the individual plasma NoA concentrations at the end of the experiment. In contrast to the effect on NoA plasma concentrations, at the end of the experiment adrenaline levels were within or even lower than the detection level. NOx and troponin-I plasma levels were 2- and fourfold higher at the end of the experiment than at baseline, whereas plasma isoprostane and both whole blood O_2_^⋅^¯ and the "tail moment" in the "comet assay" did not show any further effect. Figure 1 demonstrates that while the terminal plasma NOx concentrations (Fig. 1a) were directly related to the simultaneously obtained plasma NoA levels (NOx: r = 0.519, p = 0.033), whole blood O_2_^⋅^¯ (Fig. 1b) were not significantly related (O_2_^⋅^¯: r = 0.342, p = 0.179) to the NoA levels. No significant relationship either was found between the terminal plasma isoprostane levels nor the "tail moment" in the "comet assay" when plotted as a function of the simultaneously measured plasma NoA concentrations (r = −0.329, p = 0.182, and r = −0.192, p = 0.530, respectively; graphs not shown).

### Cardiac tissue mitochondrial respiration, noradrenaline, and nitric oxide

Neither the activities of the complexes I and II of the mitochondrial respiratory chain nor OxPhos or ETC showed any significant relation to the terminal plasma NoA (Complex I: r = − 0.208, p = 0.424 (see Fig. 3a); Complex II: r = − 0.105, p = 0.688; OxPhos: r = − 0.027, p = 0.919; ETC: r = − 0.106, p = 0.684, respectively, graphs not shown) or NOx levels (Complex I: r = − 0.026, p = 0.924; Complex II: r = 0.371, p = 0.158; OxPhos: r = 0.392, p = 0.133; ETC: r = 0.294, p = 0.270, respectively; graphs not shown).

### Cardiac tissue mitochondrial respiration, noradrenaline, nitric oxide, and adrenergic receptor expression

**Fig. 2:**
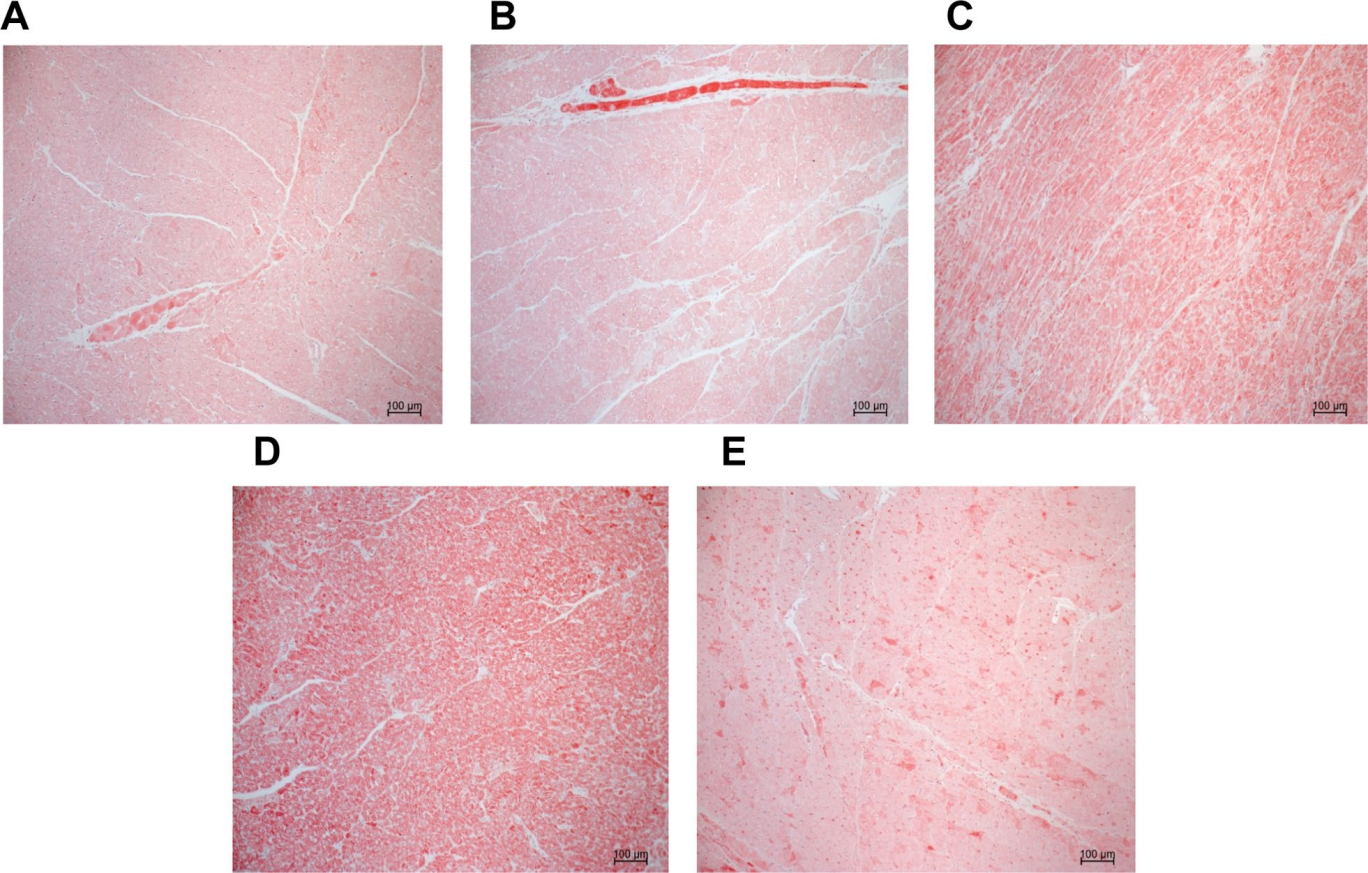
Typical immune-histochemistry images of the cardiac tissue expressions of the β_1_- (**a**) and β_2_-adrenergic (**b**) receptor, of the Complexes I (**c**) and II (**d**) as well as nitrotyrosine formation (**e**). For data on quantitative analysis, please see supplemental Table 2

**Fig. 3:**
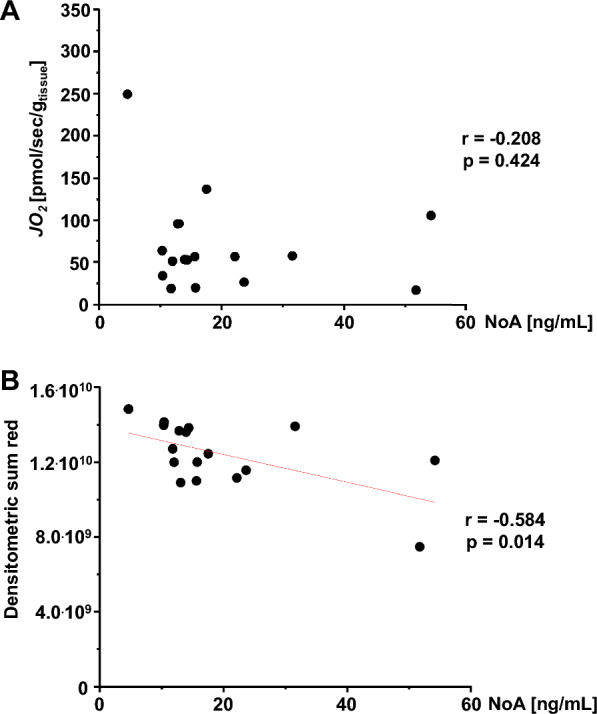
Immediate *post mortem* cardiac tissue mitochondrial *activity* (J*O*_*2*_ [pmol/sec/mg_tissue_]) (**a**) and *expression* (densitometric sum red, a dimensionless number) (**b**), of the mitochondrial complex I, plotted as a function of the plasma noradrenaline (NoA) levels ([ng/mL]) obtained immediately before the individual animal was euthanized. While no significant relation was found between Complex I *activity* and the NoA levels (r = − 0.208, p = 0.424), Complex I *expression* showed a significant inverse linear relation to NoA levels (r = − 0.584, Densitometric sum red = 1.39 · 10^10^—7.45·10^7^· NoA, p = 0.014)

**Fig. 4:**
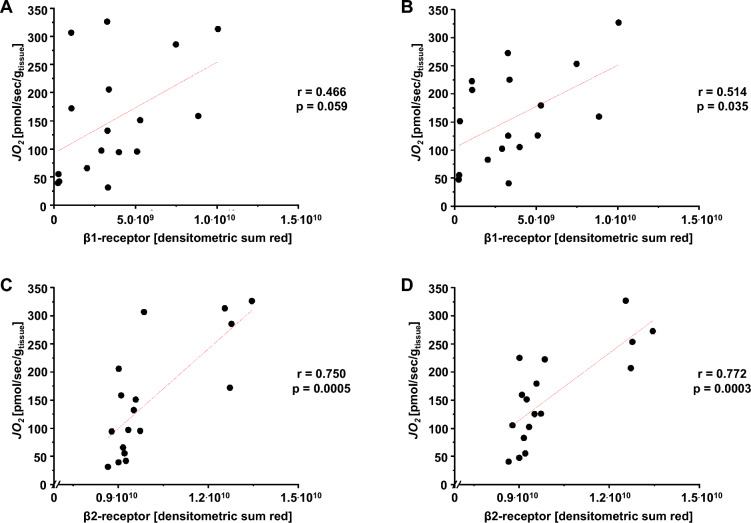
Immediate *post mortem* cardiac tissue oxidative phosphorylation (OxPhos; J*O*_*2*_ [pmol/sec/mg_tissue_]) (**a**, **c**) and maximal electron transfer capacity in the uncoupled state (ETC; J*O*_*2*_ [pmol/sec/mg_tissue_]) (**b**, **d**) plotted as a function of the β_1_- (**a**, **b**) and β_2_- (**c**, **d**) adrenergic receptor expression, respectively (densitometric sum red, a dimensionless number). While the direct linear correlation between OxPhos and the β_1_-receptor expression (r = 0.466, OxPhos = 1.62 ·10^–8^· β_1_-receptor expression + 92, p = 0.059; **a**) just missed statistical significance (p = 0.059), there were significant linear correlations between ETC and the β_1_-receptor expression ETC (r = 0.514, ETC = 1.46 ·10^–8^· β_1_-receptor expression + 104, p = 0.035; **b**) as well as both OxPhos (r = 0.750, OxPhos = 4.73 ·10^–8^· β_2_-receptor expression—326, p = 0.0005; **c**) and ETC (r = 0.772, ETC = 3.89 ·10^–8^· β_2_-receptor expression—245, p = 0.0003; **d**) and the β_2_-receptor expression

Figure 2 shows representative examples and supplemental Table 2 the quantitative analysis of the cardiac tissue expressions of the β_1_- (Fig. 2a) and β_2_-adrenergic (Fig. 2b) receptor as well as Complex I (Fig. 2c) and II (Fig. 2d) and nitrotyrosine formation (Fig. 2e). Figure 3 demonstrates that Complex I expression was inversely related to the terminal NoA concentrations (Fig. 3B; r = − 0.584, p = 0.014). In contrast, Complex II expression did not show any relation to the NoA concentrations (r = 0.071, p = 0.785; graph not shown). Tissue nitrotyrosine expression was unrelated to the plasma NOx levels as well (r = − 0.133, p = 0.637, graph not shown). Figure 4 shows that cardiac tissue OxPhos and ETC were directly related to both the tissue β_1_- (OxPhos: r = 0.466, p = 0.059, Fig. 4A; ETC: r = 0.514, p = 0.035, Fig. 4b) and β_2_-adrenergic receptor expression (OxPhos: r = 0.750, p = 0.0005, Fig. 4C; ETC: r = 0.772, p = 0.0003, Fig. 4d). Cardiac tissue expressions of the β_1_-and β_2_-adrenergic receptors were unrelated to the final NoA plasma concentrations (β_1_: r = 0.203, p = 0.435; β_2_: r = 0.139, p = 0.594, graphs not shown) and the cardiac tissue nitrotyrosine expression (β_1_: r = 0.087, p = 0.750; β_2_: r = 0.190, p = 0.480; graphs not shown). There was no relation either between any parameter of cardiac tissue mitochondrial activity and the tissue nitrotyrosine expression (OxPhos: r = 0.156, p = 0.563; ETC: r = 0.212, p = 0.432; Complex I: r = 0.272, p = 0.308; Complex II: r = 0.136, p = 0.615; graphs not shown).

### Cardiac injury and tissue mitochondrial respiration, noradrenaline, and nitric oxide

**Fig. 5:**
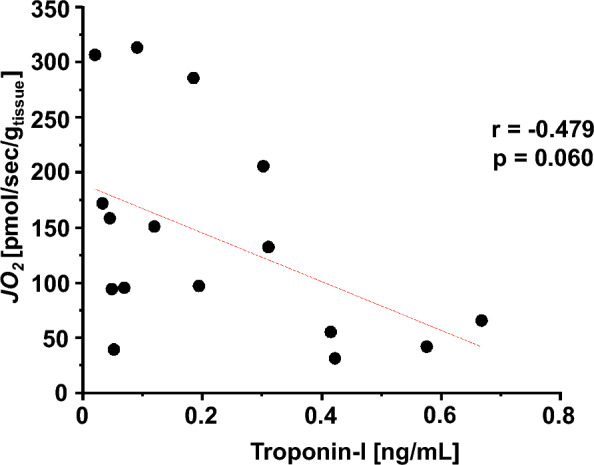
Immediate *post mortem* cardiac tissue OxPhos (J*O*_*2*_ [pmol/sec/mg_tissue_]) plotted as a function of the plasma troponin-I levels ([ng/mL]) obtained immediately before the individual animal was euthanized. There was an inverse linear correlation between OxPhos (r = − 0.479, OxPhos = − 221 · troponin-I + 189), which just missed statistical significance (p = 0.060)

Figure 5 shows that cardiac tissue OxPhos was inversely related to the plasma troponin-I concentrations (r = − 0.479, p = 0.060), whereas no association was found for ETC when plotted as a function of plasma troponin-I (r = − 0.371, p = 0.157, graph not shown). No significant relation was found either when troponin-I levels were plotted as a function of the mean NoA infusion rate and the plasma NoA or NOx levels (NoA infusion rate: r = 0.095, p = 0.726; NoA concentrations: r = 0.033, p = 0.901; NOx levels: r = − 0.039, p = 0.886; graphs not shown).

## Discussion

In our well-established long-term porcine model of combined acute subdural hematoma and hemorrhage with subsequent resuscitation [14, 15], the aim of the present study was to test the hypothesis whether there is any significant relation between immediate *post mortem* cardiac tissue mitochondrial respiration on the one hand, and the NoA plasma levels obtained immediately before euthanization on the other hand. Thereby, we sought to answer the question whether we could confirm the inverse relationship between myocardial mitochondrial respiratory activity and plasma noradrenaline concentrations within the *physiological* range under conditions of pharmacological NoA plasma levels, i.e., during continuous i.v. NoA to achieve systemic hemodynamic targets. In addition, we also investigated the putative role markers of oxidative and/or nitrosative stress as well as that of the expression of myocardial β-adrenergic receptors and the mitochondrial complex proteins. The main results were that cardiac tissue mitochondrial respiratory activity (1) did not show any significant relation to the terminal NoA and NOx concentrations or the cardiac tissue nitrotyrosine formation, whereas it was (2) directly related to both the β_1_- and β_2_-adrenergic receptor expression, and (3) inversely related to plasma troponin-I concentrations. Furthermore, while (4) plasma NOx concentrations were directly related to the simultaneously obtained plasma NoA levels, (5) neither whole blood O_2_^⋅^¯ nor plasma troponin-I levels or cardiac nitrotyrosine formation showed any association with the plasma NoA concentrations.

Baseline median plasma NoA and adrenaline concentrations [70 and 134 pg/mL, respectively] were within the same range as those in our previous experiment using animals of the same strain and with comparable size and age [16], hence, except for a single adrenaline level, all individual baseline values were below the upper threshold of the normal range reported for pigs (800 and 300 [pg/mL], respectively) [13, 29]. These values also well agree with those reported for both anesthetized and mechanically ventilated pigs under baseline conditions [13, 32] as well as awake, "non-stress susceptible" individual swine prior to transport stress [13, 33]. Hence, albeit recorded in anesthetized, mechanically ventilated animals that had undergone major (neuro) surgical instrumentation rather than in awake animals, our baseline data represent strictly physiological, un-stressed conditions. Median plasma NoA levels nearly doubled until the end of the 2 h-period of "ASDH + hemorrhage". This finding most likely mirrors the expected, adaptive physiological stress response to the pathological challenge, but this increase of the NoA concentrations was much less pronounced than that reported by other authors [34–36] after various (patho)physiological challenges. In those studies, however, smaller and younger swine were studied, different anesthesia techniques were used, and the authors studied more severe stress conditions. Strikingly, in contrast to the NoA levels, adrenaline concentrations at the end of the 2-h challenge period were significantly lower than at baseline. We can only speculate about this finding, but the combined opioid/propofol anesthesia with additional continuous i.v. midazolam infusion lasting for approx. 4–5 h at the time point may have blunted the stress response. At the end of the experiment, median NoA plasma levels were ~ 200-fold higher when compared to baseline, whereas adrenaline concentrations were close to the detection limit. An increase of NoA plasma concentrations by 2–3 orders of magnitude upon continuous i.v. NoA infusion in swine has also been reported by other authors [37, 38]; moreover, our findings well agree with clinical data from our group in patients with septic shock [32, 33]: while NoA concentrations were about 10–50-fold higher than the physiological range in patients undergoing general anesthesia for elective surgery [39, 40], adrenaline plasma concentrations were even suppressed [41].

We found no significant relation at all between the cardiac mitochondrial complex I or complex II activity and plasma NoA levels, nor between tissue OxPhos or ETC and plasma NoA concentrations. This result is in clear contrast to our previous study in swine, where significant, inverse linear relationships between both cardiac tissue OxPhos and ETC and plasma NoA and adrenaline concentrations had been observed, however, in the absence of i.v. NoA infusion [13, 16]. The present findings are also in contrast to data reported by Brealey et al. on skeletal muscle mitochondrial respiration in patients with septic shock investigated within the first 24 h of ICU admission and requiring a median continuous i.v. NoA infusion rate of approx. 0.31 µg/kg/min to maintain MAP > 60 mmHg. Clearly, in our experiment NoA infusions rates were twice to three times as high as in that study; however, the mean NoA infusion rate did not allow to predict the individual terminal plasma NoA concentrations. This latter observation well agrees with previous clinical data from our group in patients with septic shock where a twofold range of NoA infusion rates coincided with a 5–10 fold range of simultaneously obtained plasma NoA concentrations [32, 33]; hence, we can only speculate on the precise plasma NoA levels in the study by Brealey et al. Brealey et al*.* measured mitochondrial respiratory activity in biopsies obtained in vivo from the *Musculus vastis lateralis*, whereas we investigated immediate *post mortem* specimen from the heart apex. It is tempting to speculate that the response of skeletal muscle to continuous i.v. NoA might differ from that of the left ventricle, in particular under conditions of continuous analgesia and sedation as needed to allow for mechanical ventilation. Finally, the patients studied by Brealey et al. had a mean age of 65 years, and about 14% of them also had underlying chronic co-morbidity, whereas we investigated sexually mature but still young ("adolescent") animals without any apparent underlying disease.

We found a significant, direct linear relation between both the terminal plasma NOx levels and the plasma NoA concentrations; in contrast, there was no relation between the whole blood O_2_^⋅^¯ concentrations and the "tail moment", the plasma isoprostane nor cardiac tissue nitrotyrosine formation when plotted as a function of the plasma NoA concentrations. The former finding well agrees with previous data in rats and dogs: coronary artery ligation-induced myocardial infarction in rats was associated with a doubling of both tissue NoA concentrations [42]. Nevertheless, our result that there was no association between NoA levels and any of the blood or tissue markers of oxidative and/or nitrosative stress is in contrast: a parallel increase was reported for both myocardial nitrotyrosine formation [42] and atrial malondialdehyde concentrations [43] with the rise in NoA levels. Theoretically, this may be referred to the fact that the above-mentioned studies only investigated the effects of endogenously increased NoA release, which results in much lower NoA levels than the exogenous administration as in our study. In fact, our data agree with the above-mentioned findings by Brealey et al. in patients with septic shock and treated with continuous i.v. NoA to maintain MAP > 65 mmHg, i.e., presumably presenting with markedly supra-physiological NoA plasma levels: these authors found that skeletal muscle NOx content was directly related to the NoA requirements [3].

Strikingly, while mitochondrial Complex I *expression* was inversely related to the plasma NoA concentrations, no significant relation at all was present for Complex I *activity*. Taken together, this finding suggests that continuous, exogenous i.v. NoA administration, at least at doses comparable to the clinical setting, not only does not impair Complex I function in terms of mitochondrial respiration, but may even increase its "yield": the higher the NoA plasma level, the lower was the Complex I expression without effect on O_2_ consumption. Clearly, at first glance, this finding is in sharp contrast to data reported by Brealey et al. in patients with septic shock: skeletal muscle Complex I activity was inversely related to the NoA requirements. Again, a different response of skeletal *vs.* cardiac muscle may account for this difference. To the best of our knowledge, none of the previous porcine studies on endotoxic or septic shock that integrated standard ICU measures, in particular continuous i.v. NoA (≈ 0.25 up to a maximum of ≈ 2 µg/kg/min), showed any major detrimental effect on liver [9–11], skeletal [10, 11], kidney and myocardial [12] mitochondrial respiratory activity. Finally, our results corroborate findings that acute increases in plasma catecholamine levels may in fact coincide with even increased mitochondrial respiratory enzyme activity [44], e.g., during physical exercise [45].

In our experiment, plasma NOx levels showed a twofold increase over time, which is comparable to a previous experiment in swine with fecal peritonitis and resuscitated with fluids and continuous i.v. NoA and dobutamine to achieve MAP > 65 mmHg [46]. Nevertheless, plasma NOx levels did not show any significant relation to any of the other parameters measured, in particular neither to blood markers of oxidative stress or tissue nitrosative stress, i.e., nitrotyrosine expression. In contrast, Brealey et al*.* showed in rats resuscitated with fluids after fecal peritonitis that the more severe the sepsis or septic shock, the higher were NOx concentrations and the lower were both glutathione levels and Complex I and IV activity in the liver and skeletal muscle [47]. Unfortunately, the respective data on the heart were not reported, and, moreover, animals did not receive NoA for hemodynamic support.

Similar to the lacking relation between the parameters of cardiac tissue mitochondrial respiration and plasma NoA levels, we did not find any relation either between the cardiac β_1_- and β_2_-adrenergic receptor expression and plasma NoA concentrations. Hence, we could not confirm the reduced myocardial β_1_-adrenocepor receptor expression reported after long-term [4–6] and/or high-dose (200–300 μg/kg/h in rats [4, 6], 30 μg/kg/h in dogs [5]) NoA administration. However, other authors had reported β-adrenoceptor desensitization despite unchanged receptor density upon 200 µg/kg/h of NoA over up to 12 h in rats [7]. Clearly, in this context, the effects of NoA infusion may be dose-dependent: even after three months, a continuous i.v. NoA infusion in dogs, which did not produce arterial hypertension (≈ 3 μg/kg/h), both β_1_-adrenocepor receptor expression and responsiveness were even increased [8]. Moreover, a 3 mg/kg NoA i.p. bolus in rats was even associated with increased total myocardial β_1_-adrenoceptor density [2]. Finally, at least with respect to the responsiveness of cardiac mitochondrial respiration, we did not find evidence either for β-adrenergic receptor desensitization: cardiac OxPhos and ETC were directly related to both the β_1_- and β_2_-adrenergic receptor expression, indicating that the response to stimulation was preserved.

Troponin-I plasma levels, i.e., a marker of myocardial injury, progressively increased during the experiment. Since the troponin-I concentrations were neither related to the mean NoA infusion rate needed to achieve hemodynamic targets nor to the plasma NoA concentrations, we can only speculate on the role of the NoA infusion in this context. Nevertheless, our observation is in line with data from swine with lateral fluid percussion-induced traumatic brain injury: infusing phenylephrine to maintain CCP ~ 55–60 mm Hg coincided with nearly doubled plasma troponin-I concentrations [48]. At first glance, the present findings are in contrast to a previous study of our group [24]: in that study, therapeutic hyperoxia in the acute phase of resuscitation from hemorrhagic shock had been associated with lower troponin-I concentrations but unchanged tissue OxPhos. It should be noted, however, that NoA requirements to achieve hemodynamic targets had not differed either, and, in particular, in that study we had used swine with underlying coronary artery disease. Moreover, our findings that there was no association between the plasma troponin and NoA concentrations, and the simultaneous observation that there was an inverse relation between cardiac OxPhos and plasma troponin-I levels agree with recent data from a porcine study targeting cardiac ischemia‑reperfusion injury after hemorrhagic shock [49]: elamipretide reduced troponin levels and preserved cardiac mitochondrial ultrastructure, while NoA requirements remained unaffected.

## Conclusion

In a clinically relevant, long-term, resuscitated porcine model of combined acute subdural hematoma and hemorrhage, we investigated the possible impact of increased NoA plasma levels resulting from continuous i.v. NoA infusion on cardiac mitochondrial respiratory activity and the expression of mitochondrial complex proteins and β-adrenergic receptors blood as well as on blood and cardiac tissue markers of radical production, oxidative and/or nitrosative stress and cardiac injury. While both whole blood O_2_^⋅^¯ and plasma NOx concentrations correlated with NoA plasma levels, neither mitochondrial respiration nor markers of oxidative and nitrosative stress showed any relation to the NoA concentrations. β_1_- and β_2_-adrenergic receptor expressions were not related to the NoA levels either, whereas mitochondrial respiration was correlated to the β_1_- and β_2_-adrenergic receptor expression. Cardiac tissue OxPhos was inversely related to the plasma troponin-I concentrations. Hence, under conditions of *pharmacological* NoA plasma levels due to continuous i.v. NoA, we could not confirm the previously shown inverse relationship between myocardial mitochondrial respiratory activity and plasma NoA concentrations within the *physiological* range. Consequently, our results suggest that impaired cardiac tissue mitochondrial respiration during continuous i.v. noradrenaline may be absent during resuscitation from trauma-and-hemorrhage. Nevertheless, cardiac tissue injury seems to be associated with reduced mitochondrial respiration.

## Supplementary Information

Additional file1

## Data Availability

The datasets used and/or analyzed during the current study are available from the corresponding author on reasonable request.
