# Supplementary material for: Impact of noradrenaline, adrenergic receptors, and radical formation on cardiac mitochondria in resuscitated swine
Source: Intensive Care Med Exp. 2026 Jun 9;14:70. doi: 10.1186/s40635-026-00925-1 (PMC13250008; doi:10.1186/s40635-026-00925-1)
Supplement: Supplementary file 1 — Additional file1 [file 40635_2026_925_MOESM1_ESM.docx]

**Supplemental figure 1** Plasma noradrenaline (NoA) concentrations ([ng/mL]) at the end of the experiment, i.e., at 48 hours of ICU care, plotted as a function of the mean NoA infusion rate ([µg/kg/min]) needed to achieve hemodynamic targets. There was a significant linear correlation between the NoA concentrations and the infusion rate (r = 0.638, concentration = 17.5 · infusion rate + 6.9; p = 0.006).


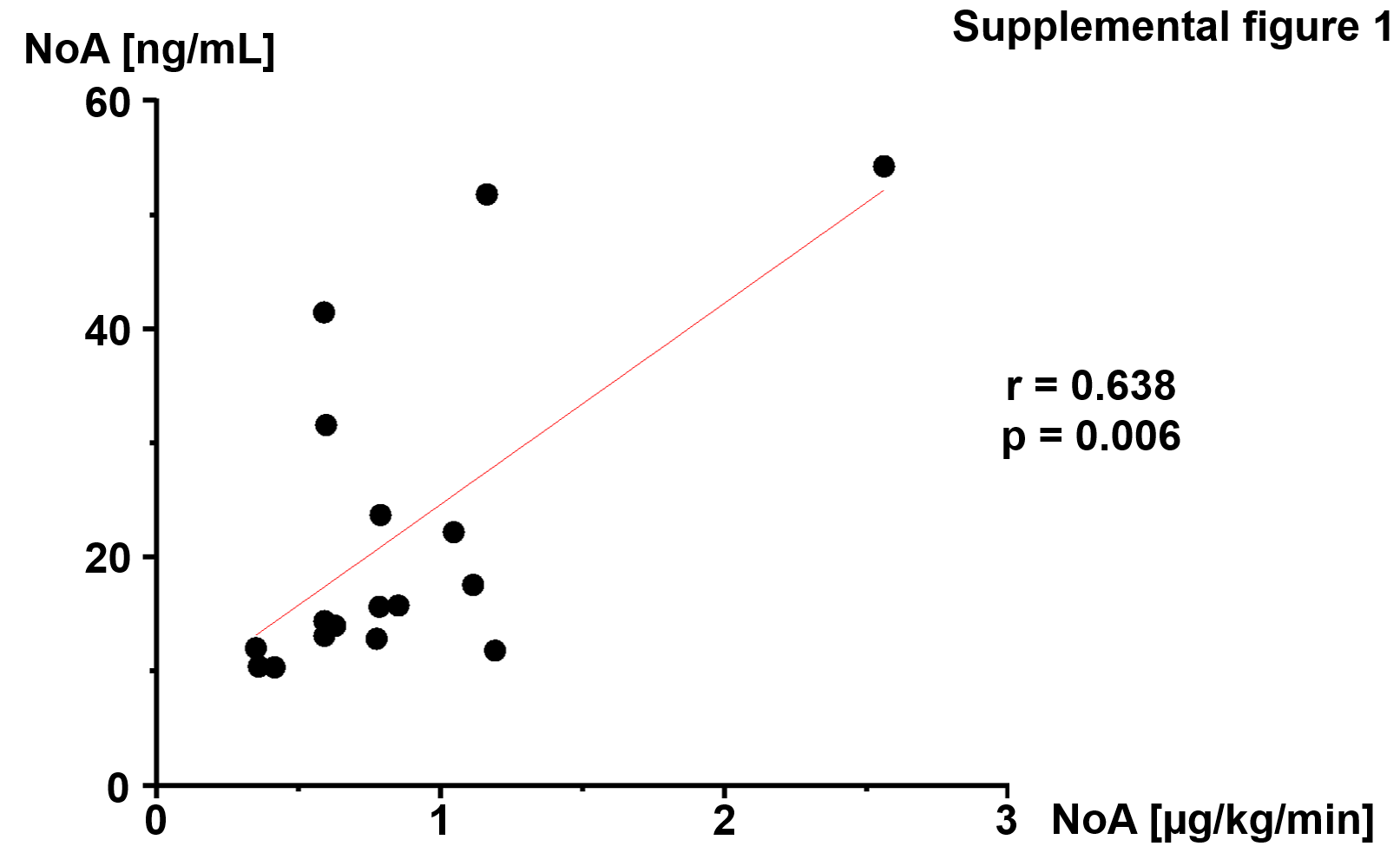


|  | Baseline | 2 hours ASDH + hemorrhage | 48 hours ICU |
| --- | --- | --- | --- |
| ICP right [mmHg] | 15 (10; 19) | 18 (12; 19) | 19 (17; 25) # |
| CPP right [mmHg] | 85 (80; 95) | 50 (38; 59) # | 89 (78; 101) |
| P_bt_O_2_ right [mmHg] | 3 (1; 25) | 2 (1; 5) | 20 (15; 33) # |
| Temperature right [°C] | 37.1 (36.8; 37.49) | 36.9 (36.7; 37.8) | 38.5 (37.9; 38.7) # |
| ICP left [mmHg] | 12 (9; 16) | 12 (10; 19) | 19 (15; 25) # |
| CPP left [mmHg] | 87 (80; 91) | 49 (43; 64) # | 90 (80; 102) |
| P_bt_O_2_ left [mmHg] | 5 (2; 16) | 2 (1; 15) | 31 (19; 42) # |
| Temperature left [°C] | 37.0 (36.7; 37.3) | 36.9 (36.6; 37.4) | 38.6 (37.7; 38.8) # |

**Supplemental Table 1.** Brain tissue temperature as well as parameters of brain perfusion (ICP intracranial pressure, CPP cerebral perfusion pressure) and oxygenation (P_bt_O_2_ brain tissue oxygen partial pressure) before ("Baseline"), immediately after combined acute subdural hematoma and hemorrhage ("2 hours ASDH + hemorrhage"), as well as at 48 hours of intensive care ("ICU"). "Left" and "right" refer to the blood-injected and sham-instrumented brain hemispheres, respectively. Data is presented as median (interquartile range), # denotes p < 0.05 vs. "Baseline".

| β_1_-adrenergic receptor | 3.29 (1.08 – 5.10) · 10^9^ |
| --- | --- |
| β_2_-adrenergic receptor | 9.33 (9.10 – 9.86) · 10^9^ |
| Mitochondrial complex 1 | 1.25 (1.16 – 1.38) · 10^10^ |
| Mitochondrial complex 2 | 8.99 · 10^9^ (6.71 · 10^9^ - 1.13 · 10^10^) |
| Nitrotyrosine | 1.05 · 10^10^ (7.47 · 10^9^ – 1.32 · 10^10^) |

**Supplemental Table 2.** Quantitative analysis of the immune histochemistry analyses in immediate *post mortem* cardiac tissue specimen. All data is presented as median (interquartile range) of the densitometric sum red.
